# Supplementary material for: Differences in Atrial Remodeling in Hypertrophic Cardiomyopathy Compared to Hypertensive Heart Disease and Athletes’ Hearts
Source: J Clin Med. 2022 Feb 27;11(5):1316. doi: 10.3390/jcm11051316 (PMC8910879; doi:10.3390/jcm11051316)
Supplement: Supplementary file 1 [file jcm-11-01316-s001.zip › jcm-1602320-supplementary.pdf]

Differences in atrial remodeling in hypertrophic cardiomyopathy compared to hypertensive heart disease and athletes' hearts

Helge Servatius, MD, Simon Raab, MD, Babken Asatryan, MD, PhD, Andreas Haeberlin, MD, PhD, Mattia Branca, PhD, Stefano de Marchi, MD, Nicolas Brugger, MD, Nikolas Nozica, MD, Eleni Goulouti, MD, Elena Elchinova, MD, PhD, Anna Lam, MD, Jens Seiler, MD, Fabian Noti, MD, Antonio Madaffari, MD, Hildegard Tanner, MD, Samuel H. Baldinger, MD, Tobias Reichlin, MD, Matthias Wilhelm, MD, Laurent Roten, MD

Supplementary Material

Table S1. Clinical characteristics of patient subgroups with interventricular septum thickness ≥13 mms in HHD group and athletes.

	HCM N=27	HHD N=114	P value*	Athletes N=12	P value**
**Clinical characteristics**					
Age, years	50 ± 14	76 ± 5.6	<0.001	43 ± 10	0.148
Sex, female	8 (30%)	49 (43%)	0.277	-	-
BMI, kg/m2	27 ± 4.8	28 ± 4.2	0.253	24 ± 2.2	0.022
Arterial hypertension	10 (37%)	115 (100%)	<0.001	-	-
Diabetes mellitus	4 (15%)	37 (32%)	0.100	-	-
Dyslipidemia	11 (41%)	80 (70%)	0.006	-	-
Coronary artery disease	2 (7%)	53 (46%)	<0.001	-	-
Congestive heart failure	-	4 (3%)	1.000	-	-
Previous thrombotic event	2 (7%)	21 (18%)	0.247	-	-
Medication					
Betablocker	14 (52%)	52 (45%)	0.669	-	-
Calcium channel blocker	9 (33%)	36 (31%)	1.000	-	-
ACE inhibitors	5 (19%)	32 (28%)	0.343	-	-
ARB	3 (11%)	46 (40%)	0.003	-	-
Aldactone	1 (4%)	3 (3%)	0.583	-	-
Diuretics	4 (15%)	27 (23%)	0.440	-	-
Statins	10 (37%)	79 (69%)	0.003	-	-
**ECG**					
Heart rate, bpm	63 ± 11	69 ± 10	0.008	55 ± 9.0	0.026
PR interval, ms	191 ± 48	181 ± 31	0.160	164 ± 17	0.058
QRS width, ms	110 ± 27	97 ± 20	0.007	99 ± 8.8	0.166
QTc, ms	448 ± 27	439 ± 26	0.128	411 ± 17	<0.001
**24-hour Holter ECG**					
Minimal heart rate, beats per minute	54 ±8.4	55 ± 8.0	0.296	42 ± 5.6	0.010
Number of PACs per hour	4.9 ± 16	17 ± 38	0.001	0.2 ± 0.3	0.623
**SAECG**					
Filtered P wave duration, ms	153 ± 26	145 ± 17	0.056	135 ± 6.7	0.041
RMS voltage of P wave, µV	7.5 ± 2.4	6.5 ± 2.4	0.047	8.2 ± 1.7	0.393
P wave integral, µVs	850 ± 272	673 ± 250	0.001	835 ± 183	0.874
RMS voltage of terminal 20 ms, µV	3.8 ± 1.4	4.4 ± 2.1	0.194	4.3 ± 1.8	0.286
RMS voltage of terminal 30 ms, µV	3.9 ± 1.3	4.4 ± 2.2	0.196	4.3 ± 1.8	0.409
RMS voltage of terminal 40 ms, µV	5.2 ± 1.8	5.2 ± 2.5	0.912	6.0 ± 3.3	0.367
**Laboratory**					
BNP, pg/mL	142 ± 126	86 ± 88	0.007	-	-
hsTNT, µg/L	0.02 ± 0.02	0.02 ± 0.02	0.805	-	-
hsCRP, mg/L	2.8 ± 4.0	3.1 ± 3.9	0.712	-	-
**Echocardiography**					
LVEF, %	66 ± 7.0	62 ± 5.5	0.001	67 ± 4.5	0.731
LVEDD, mm	44 ± 6.4	47 ± 6.8	0.110	50 ± 4.1	0.004
IVS, mm	18 ± 3.4	14 ± 1.3	<0.001	13 ± 0.7	<0.001
PW, mm	11 ± 3.3	12 ± 1.9	0.702	11 ± 1.1	0.630
LVMI, g/m^2	154 ± 56	143 ± 32	0.208	123 ± 18	0.069
RVD, mm	32 ± 5.7	32 ± 5.0	0.723	37 ± 5.6	0.032
RV TDI S, cm/s	13 ± 2.8	14 ± 3.1	0.157	15 ± 3.5	0.042
RV diastolic area, cm^2	18 ± 3.5	16 ± 5.3	0.322	23	0.060
LAVI, ml/m^2	43 ± 14	32 ± 12	<0.001	16 ± 2.2	<0.001
E wave, cm/s	71 ± 20	67 ± 19	0.352	73 ± 15	0.674
A wave, cm/s	62 ± 25	89 ± 20	<0.001	50 ± 8.7	0.122
E wave / A wave ratio	1.4 ± 1.0	0.8 ± 0.3	<0.001	1.5 ± 0.3	0.807

Isovolumetric relaxation time, ms	101 ± 13	98 ± 26	0.685	84 ± 10	0.001
E deceleration time, ms	222 ± 60	270 ± 64	<0.001	180 ± 25	0.024
E wave TDI, cm/s	5.8 ± 1.8	5.6 ± 1.7	0.440	10 ± 2.2	<0.001
E wave / E wave TDI ratio	14 ± 10	13 ± 5.1	0.508	7.5 ± 2.2	0.033
A wave TDI, cm/s	7.2 ± 2.1	10 ± 2.1	<0.001	9.4 ± 1.7	0.003

Shown are numbers with percentages in parentheses, or means ± standard deviations, as appropriate. *Comparing HCM versus HHD patients. **Comparing HCM patients versus athletes. ACE: angiotensin converting enzyme; ARB: angiotensin receptor blocker; BMI: body mass index; BP: blood pressure; CAD: coronary artery disease; CCB: calcium channel blocker; ECG: electrocardiogram; HCM: hypertrophic cardiomyopathy; HHD: hypertensive heart disease; PACS: premature atrial contractions; RMS: root-mean-square; SAECG: signal-averaged ECG; PW: posterior wall; LVMI: left ventricular mass index; RVD: right ventricular diameter.
